# Supplementary material for: The Group Contribution to the Function Derived from Density and Speed-of-Sound Measurements for Glymes in N,N-Dimethylformamide + Water Mixtures
Source: Molecules. 2023 Feb 4;28(4):1519. doi: 10.3390/molecules28041519 (PMC9968067; doi:10.3390/molecules28041519)
Supplement: Supplementary file 1 [file molecules-28-01519-s001.zip › molecules-2185925-supplementary.pdf]

The group contribution to the function derived from density and speed of sound measurements for glymes in the *N,N*-dimethylformamide + water mixtures.

Małgorzata Jóźwiak[a*], Marlena Komudzińska[a], Magdalena Tyczyńska[a]
[a]Department of Physical Chemistry, Faculty of Chemistry, University of Lodz, Pomorska 165, 90-236 Lodz, Poland
[*]corresponding author: malgorzata.jozwiak@chemia.uni.lodz.pl

Supplementary Materials

Table S1. Density of the pentaglyme + DMF + W solution at pressure p = 0.100 ± 0.005MPa.

		$\rho/(\mathrm{kg\cdot m^{-3}})$			
	m/mol·kg^{-1}	293.15 K	298.15 K	303.15 K	308.15 K
x_W = 1.000	0.00000	998.204	997.050	995.659	994.039
	0.02720	998.914	997.734	996.316	994.674
	0.04899	999.482	998.280	996.841	995.180
	0.07406	1000.132	998.906	997.442	995.758
	0.12010	1001.321	1000.049	998.535	996.812
	0.17162	1002.640	1001.320	999.748	997.978
	0.19593	1003.261	1001.914	1000.316	998.523
	0.23202	1004.177	1002.793	1001.155	999.326
	0.26652	1005.047	1003.631	1001.950	1000.087
x_W = 0.980	0.00000	997.773	996.268	994.552	992.641
	0.02290	998.397	996.867	995.129	993.196
	0.05007	999.131	997.572	995.808	993.849
	0.07274	999.739	998.155	996.369	994.388
	0.12350	1001.084	999.445	997.611	995.578
	0.17066	1002.312	1000.624	998.743	996.663
	0.19792	1003.012	1001.298	999.390	997.280
	0.22953	1003.819	1002.070	1000.131	997.988
	0.26544	1004.723	1002.938	1000.961	998.782
x_W = 0.960	0.00000	998.064	996.187	994.128	991.899
	0.02556	998.782	996.879	994.795	992.540
	0.05031	999.470	997.540	995.433	993.152
	0.07117	1000.043	998.092	995.964	993.662

	0.12387	1001.470	999.464	997.284	994.928
	0.17126	1002.724	1000.669	998.442	996.037
	0.19723	1003.400	1001.318	999.064	996.635
	0.23223	1004.298	1002.178	999.892	997.424
	0.26541	1005.138	1002.981	1000.664	998.161
$x_W = 0.940$	0.00000	998.615	996.399	994.011	991.478
	0.02549	999.344	997.100	994.685	992.124
	0.04819	999.984	997.716	995.277	992.693
	0.07117	1000.624	998.331	995.870	993.262
	0.12085	1001.981	999.637	997.127	994.469
	0.17119	1003.319	1000.926	998.368	995.659
	0.19426	1003.920	1001.503	998.925	996.194
	0.23011	1004.837	1002.389	999.778	997.014
	0.26290	1005.665	1003.188	1000.546	997.751
$x_W = 0.920$	0.00000	999.220	996.662	993.975	991.159
	0.02543	999.937	997.353	994.639	991.797
	0.04882	1000.588	997.981	995.241	992.376
	0.07199	1001.225	998.594	995.830	992.942
	0.12158	1002.560	999.880	997.065	994.128
	0.17003	1003.831	1001.101	998.237	995.252
	0.19963	1004.587	1001.829	998.935	995.922
	0.23678	1005.521	1002.724	999.793	996.747
	0.26349	1006.180	1003.354	1000.403	997.328
$x_W = 0.900$	0.00000	999.732	996.885	993.911	990.832
	0.02802	1000.495	997.617	994.616	991.512
	0.05067	1001.103	998.201	995.179	992.054
	0.06925	1001.595	998.675	995.634	992.494
	0.12312	1002.997	1000.021	996.930	993.742
	0.16960	1004.170	1001.153	998.017	994.790
	0.19731	1004.853	1001.813	998.650	995.399
	0.23046	1005.657	1002.586	999.396	996.118
	0.26704	1006.526	1003.429	1000.199	996.896
$x_W = 0.800$	0.00000	999.831	995.952	992.012	988.006

	0.02662	1000.407	996.510	992.554	988.532
	0.05278	1000.963	997.049	993.077	989.041
	0.07110	1001.347	997.421	993.438	989.393
	0.12147	1002.379	998.422	994.410	990.341
	0.17142	1003.368	999.381	995.342	991.253
	0.19612	1003.844	999.843	995.793	991.694
	0.22873	1004.459	1000.440	996.375	992.263
	0.26881	1005.197	1001.155	997.072	992.953
x_W = 0.700	0.00000	995.512	991.156	986.765	982.306
	0.02926	996.023	991.658	987.258	982.790
	0.05503	996.464	992.090	987.683	983.208
	0.07243	996.756	992.378	987.965	983.485
	0.12016	997.539	993.146	988.721	984.228
	0.17029	998.328	993.921	989.484	984.977
	0.19564	998.714	994.300	989.857	985.346
	0.23316	999.270	994.846	990.395	985.877
	0.26766	999.767	995.333	990.874	986.348
x_W = 0.600	0.00000	988.573	984.000	979.401	974.763
	0.02648	988.985	984.406	979.803	975.159
	0.04971	989.340	984.756	980.148	975.499
	0.07105	989.659	985.071	980.460	975.807
	0.12025	990.374	985.777	981.159	976.496
	0.17040	991.074	986.465	981.840	977.170
	0.19550	991.412	986.800	982.172	977.498
	0.23012	991.866	987.250	982.617	977.939
	0.26635	992.328	987.708	983.070	978.389
x_W = 0.500	0.00000	980.846	976.193	971.509	966.803
	0.02201	981.178	976.521	971.833	967.124
	0.05108	981.608	976.946	972.254	967.540
	0.07481	981.954	977.286	972.592	967.873
	0.12232	982.627	977.953	973.251	968.525
	0.17008	983.280	978.600	973.889	969.159
	0.19646	983.631	978.947	974.232	969.499

	0.23312	984.105	979.417	974.699	969.963
	0.27166	984.592	979.899	975.179	970.438
x_W = 0.400	0.00000	973.014	968.325	963.609	958.847
	0.02618	973.424	968.729	964.009	959.244
	0.05053	973.798	969.099	964.374	959.608
	0.07297	974.137	969.434	964.706	959.937
	0.12102	974.845	970.135	965.400	960.625
	0.17060	975.549	970.834	966.093	961.309
	0.19577	975.897	971.179	966.435	961.647
	0.23433	976.416	971.696	966.944	962.154
	0.27634	976.967	972.244	967.489	962.691
x_W = 0.300	0.00000	965.715	960.997	956.255	951.499
	0.02246	966.100	961.380	956.635	951.876
	0.05194	966.597	961.874	957.125	952.362
	0.07419	966.966	962.241	957.489	952.723
	0.12146	967.731	963.001	958.244	953.474
	0.16973	968.488	963.752	958.991	954.217
	0.19491	968.871	964.133	959.370	954.595
	0.23015	969.397	964.656	959.890	955.113
	0.27596	970.063	965.318	960.551	955.769
x_W = 0.200	0.00000	959.317	954.581	949.829	945.053
	0.02621	959.845	955.104	950.348	945.570
	0.04975	960.310	955.566	950.807	946.027
	0.07225	960.749	956.001	951.239	946.458
	0.12419	961.733	956.980	952.212	947.428
	0.16946	962.562	957.803	953.034	948.244
	0.19216	962.965	958.206	953.438	948.648
	0.23052	963.634	958.873	954.103	949.313
	0.27650	964.417	959.651	954.883	950.086
x_W = 0.100	0.00000	953.743	948.988	944.227	939.448
	0.02383	954.311	949.552	944.788	940.006
	0.05002	954.921	950.159	945.392	940.606
	0.07364	955.461	950.694	945.924	941.136

	0.12204	956.528	951.755	946.979	942.187
	0.17099	957.557	952.779	948.000	943.201
	0.19562	958.056	953.275	948.493	943.692
	0.23233	958.776	953.993	949.210	944.407
	0.27585	959.603	954.806	950.025	945.227
x_W = 0.000	0.00000	948.744	943.979	939.207	934.428
	0.02386	949.390	944.623	939.849	935.071
	0.05145	950.114	945.343	940.568	935.791
	0.07363	950.677	945.905	941.129	936.353
	0.12070	951.816	947.043	942.268	937.491
	0.17089	952.946	948.176	943.404	938.627
	0.19481	953.456	948.689	943.917	939.139
	0.23328	954.236	949.470	944.706	939.928
	0.27780	955.072	950.317	945.558	940.776

The uncertainty of the mole fraction x_W is equal to $\pm 1\cdot 10^{-3}$. The uncertainty of molality is equal to $\pm 1.5\cdot 10^{-5}$ mol·kg^{-1}.

Table S2. Density of the hexaglyme + DMF + W solution at pressure p = 0.100 ± 0.005MPa.

	$\rho / (\mathrm{kg\cdot m^{-3}})$				
	m/mol·kg^{-1}	293.15 K	298.15 K	303.15 K	308.15 K
x_W = 1.000	0.00000	998.208	997.055	995.653	994.043
	0.02654	999.043	997.859	996.428	994.790
	0.04718	999.690	998.483	997.027	995.367
	0.07348	1000.508	999.273	997.782	996.096
	0.12072	1001.966	1000.676	999.131	997.394
	0.16577	1003.341	1001.995	1000.403	998.614
	0.19290	1004.162	1002.781	1001.155	999.342
	0.23377	1005.389	1003.960	1002.284	1000.426
	0.26703	1006.382	1004.911	1003.194	1001.298
x_W = 0.980	0.00000	997.775	996.263	994.558	992.646
	0.02630	998.677	997.134	995.400	993.461
	0.04688	999.377	997.809	996.053	994.091
	0.07142	1000.204	998.606	996.824	994.836

	0.11931	1001.796	1000.138	998.304	996.266
	0.17341	1003.557	1001.833	999.939	997.845
	0.19399	1004.218	1002.468	1000.548	998.436
	0.22823	1005.303	1003.515	1001.562	999.408
	0.26482	1006.445	1004.618	1002.625	1000.427
x_W = 0.960	0.00000	998.068	996.182	994.125	991.897
	0.02806	999.058	997.136	995.044	992.786
	0.04993	999.821	997.871	995.752	993.470
	0.06868	1000.468	998.495	996.353	994.050
	0.11870	1002.166	1000.131	997.928	995.573
	0.16749	1003.782	1001.687	999.430	997.025
	0.19471	1004.665	1002.542	1000.251	997.819
	0.23961	1006.108	1003.926	1001.583	999.103
	0.26936	1007.045	1004.828	1002.452	999.943
x_W = 0.940	0.00000	998.618	996.396	994.007	991.473
	0.02764	999.613	997.356	994.932	992.366
	0.04917	1000.378	998.094	995.642	993.051
	0.06764	1001.025	998.718	996.243	993.632
	0.11687	1002.721	1000.352	997.817	995.153
	0.16487	1004.328	1001.902	999.307	996.595
	0.19164	1005.205	1002.747	1000.121	997.383
	0.23578	1006.619	1004.112	1001.436	998.654
	0.26501	1007.537	1005.001	1002.287	999.478
x_W = 0.920	0.00000	999.226	996.669	993.978	991.155
	0.02492	1000.108	997.519	994.800	991.950
	0.04866	1000.936	998.317	995.572	992.696
	0.07143	1001.718	999.071	996.302	993.401
	0.11787	1003.281	1000.578	997.759	994.811
	0.17385	1005.104	1002.341	999.461	996.458
	0.20086	1005.962	1003.168	1000.262	997.232
	0.22911	1006.840	1004.017	1001.085	998.028
	0.25937	1007.769	1004.910	1001.951	998.867
x_W = 0.900	0.00000	999.738	996.889	993.918	990.836

	0.02702	1000.673	997.794	994.794	991.685
	0.04911	1001.426	998.523	995.499	992.369
	0.06985	1002.125	999.198	996.152	993.003
	0.11608	1003.651	1000.673	997.580	994.386
	0.16915	1005.350	1002.316	999.169	995.928
	0.19264	1006.084	1003.027	999.856	996.594
	0.23327	1007.326	1004.229	1001.021	997.724
	0.26404	1008.247	1005.124	1001.884	998.563
x_W = 0.800	0.00000	999.839	995.958	992.017	988.009
	0.02631	1000.594	996.697	992.741	988.714
	0.05015	1001.259	997.350	993.380	989.340
	0.06965	1001.798	997.879	993.899	989.844
	0.12002	1003.158	999.212	995.205	991.114
	0.16883	1004.429	1000.458	996.428	992.302
	0.19503	1005.095	1001.108	997.067	992.924
	0.23372	1006.052	1002.046	997.986	993.818
	0.26945	1006.911	1002.889	998.812	994.618
x_W = 0.700	0.00000	995.509	991.159	986.768	982.307
	0.02721	996.154	991.794	987.392	982.922
	0.05019	996.690	992.320	987.909	983.432
	0.07042	997.154	992.776	988.358	983.874
	0.11807	998.221	993.826	989.391	984.891
	0.16552	999.247	994.836	990.386	985.870
	0.19639	999.894	995.475	991.015	986.488
	0.23262	1000.635	996.206	991.734	987.199
	0.26930	1001.371	996.930	992.449	987.900
x_W = 0.600	0.00000	988.569	984.007	979.409	974.779
	0.02582	989.123	984.556	979.953	975.318
	0.04807	989.593	985.021	980.413	975.773
	0.07046	990.058	985.481	980.869	976.225
	0.11933	991.047	986.461	981.840	977.186
	0.16988	992.033	987.438	982.807	978.146
	0.19224	992.456	987.859	983.224	978.559

	0.23002	993.158	988.552	983.912	979.243
	0.27268	993.927	989.317	984.669	979.993
x_W = 0.500	0.00000	980.844	976.191	971.506	966.804
	0.02620	981.386	976.729	972.039	967.332
	0.04908	981.851	977.190	972.496	967.786
	0.07135	982.297	977.632	972.935	968.221
	0.11924	983.233	978.560	973.855	969.133
	0.16660	984.127	979.447	974.735	970.006
	0.19558	984.659	979.975	975.257	970.525
	0.23001	985.276	980.589	975.866	971.129
	0.27069	985.986	981.296	976.570	971.824
x_W = 0.400	0.00000	973.003	968.312	963.596	958.835
	0.02533	973.554	968.857	964.139	959.374
	0.04807	974.040	969.339	964.618	959.849
	0.07303	974.566	969.860	965.136	960.363
	0.12068	975.546	970.832	966.103	961.322
	0.16770	976.481	971.762	967.025	962.238
	0.19931	977.093	972.369	967.628	962.837
	0.23383	977.749	973.021	968.274	963.478
	0.27474	978.506	973.775	969.020	964.222
x_W = 0.300	0.00000	965.713	960.995	956.254	951.497
	0.02667	966.351	961.629	956.883	952.122
	0.05097	966.923	962.197	957.446	952.682
	0.07071	967.380	962.651	957.897	953.129
	0.11946	968.482	963.746	958.985	954.211
	0.16816	969.548	964.803	960.036	955.257
	0.19414	970.099	965.352	960.583	955.800
	0.23318	970.910	966.157	961.385	956.598
	0.27451	971.747	966.991	962.212	957.422
x_W = 0.200	0.00000	959.319	954.584	949.833	945.054
	0.02406	959.984	955.245	950.489	945.706
	0.05074	960.707	955.965	951.205	946.416
	0.06912	961.198	956.453	951.690	946.899

	0.12024	962.529	957.779	953.011	948.210
	0.17291	963.850	959.092	954.323	949.514
	0.19690	964.434	959.675	954.905	950.093
	0.23388	965.315	960.553	955.780	950.965
	0.26538	966.050	961.286	956.510	951.695
x_W = 0.100	0.00000	953.742	948.985	944.229	939.449
	0.02556	954.544	949.784	945.021	940.238
	0.04951	955.278	950.514	945.747	940.961
	0.07371	956.004	951.235	946.465	941.675
	0.12028	957.354	952.574	947.798	943.005
	0.16628	958.626	953.833	949.055	944.259
	0.19362	959.354	954.553	949.774	944.977
	0.23091	960.312	955.498	950.721	945.924
	0.28006	961.514	956.682	951.909	947.112
x_W = 0.000	0.00000	948.746	943.976	939.205	934.427
	0.02672	949.687	944.913	940.138	935.358
	0.04932	950.458	945.682	940.904	936.122
	0.07293	951.238	946.462	941.679	936.898
	0.12086	952.743	947.970	943.176	938.401
	0.17102	954.206	949.440	944.633	939.871
	0.19284	954.808	950.046	945.232	940.478
	0.23187	955.827	951.078	946.250	941.514
	0.28013	956.991	952.261	947.415	942.707

The uncertainty of the mole fraction x_W is equal to $\pm 1\cdot 10^{-3}$. The uncertainty of molality is equal to $\pm 1.5\cdot 10^{-5}$ mol·kg^{-1}.

Table S3. Speed of sound in the pentaglyme + DMF + W solution at pressure p = 0.100 ± 0.005MPa.

		$u/\left(\mathrm{m\cdot s^{-1}}\right)$			
	m/(mol·kg^{-1})	293.15 K	298.15 K	303.15 K	308.15 K
x_W = 1.000	0.00000	1483.1	1497.3	1509.7	1520.3
	0.02720	1487.6	1501.4	1513.4	1523.7
	0.04899	1491.2	1504.7	1516.4	1526.4

	0.07406	1495.4	1508.4	1519.8	1529.5
	0.12010	1503.1	1515.3	1526.0	1535.2
	0.17162	1511.7	1523.0	1533.0	1541.6
	0.19593	1515.8	1526.7	1536.2	1544.7
	0.23202	1521.8	1532.1	1541.1	1549.2
	0.26652	1527.5	1537.2	1545.8	1553.5
x_W = 0.980	0.00000	1534.3	1543.2	1550.6	1556.5
	0.02290	1537.7	1546.3	1553.3	1559.0
	0.05007	1541.6	1549.9	1556.6	1561.9
	0.07274	1545.0	1552.9	1559.3	1564.3
	0.12350	1552.4	1559.8	1565.5	1569.8
	0.17066	1559.3	1566.1	1571.2	1574.9
	0.19792	1563.3	1569.7	1574.4	1577.8
	0.22953	1568.0	1574.0	1578.2	1581.2
	0.26544	1573.2	1578.8	1582.6	1585.1
x_W = 0.960	0.00000	1575.1	1579.5	1582.6	1584.7
	0.02556	1578.7	1582.7	1585.6	1587.3
	0.05031	1582.2	1585.8	1588.4	1589.8
	0.07117	1585.0	1588.4	1590.7	1591.9
	0.12387	1592.0	1594.7	1596.4	1596.9
	0.17126	1598.1	1600.2	1601.4	1601.3
	0.19723	1601.3	1603.1	1604.0	1603.6
	0.23223	1605.5	1607.0	1607.4	1606.7
	0.26541	1609.5	1610.5	1610.6	1609.5
x_W = 0.940	0.00000	1610.0	1610.0	1609.4	1608.0
	0.02549	1613.1	1612.9	1611.9	1610.1
	0.04819	1615.8	1615.4	1614.0	1612.0
	0.07117	1618.5	1617.9	1616.2	1613.9
	0.12085	1624.1	1623.2	1620.7	1617.9
	0.17119	1629.6	1628.3	1625.1	1621.7
	0.19426	1632.1	1630.6	1627.0	1623.5
	0.23011	1635.8	1634.0	1629.9	1626.0
	0.26290	1639.1	1637.1	1632.5	1628.3

x_W = 0.920	0.00000	1635.4	1632.5	1628.9	1624.7
	0.02543	1638.0	1634.8	1630.9	1626.5
	0.04882	1640.3	1636.9	1632.7	1628.1
	0.07199	1642.5	1638.9	1634.4	1629.7
	0.12158	1647.0	1642.9	1637.9	1632.8
	0.17003	1651.1	1646.5	1641.0	1635.6
	0.19963	1653.5	1648.6	1642.7	1637.1
	0.23678	1656.2	1651.0	1644.8	1639.0
	0.26349	1658.1	1652.7	1646.2	1640.2
x_W = 0.900	0.00000	1656.9	1651.2	1644.9	1638.2
	0.02802	1659.2	1653.2	1646.7	1639.7
	0.05067	1661.0	1654.8	1648.1	1640.9
	0.06925	1662.5	1656.1	1649.2	1641.9
	0.12312	1666.7	1659.8	1652.4	1644.6
	0.16960	1670.1	1662.8	1655.0	1646.9
	0.19731	1672.1	1664.5	1656.5	1648.2
	0.23046	1674.4	1666.6	1658.3	1649.7
	0.26704	1676.9	1668.7	1660.2	1651.3
x_W = 0.800	0.00000	1701.0	1688.0	1674.6	1661.0
	0.02662	1701.4	1688.3	1674.8	1661.0
	0.05278	1701.7	1688.5	1674.9	1661.0
	0.07110	1701.9	1688.6	1675.0	1661.0
	0.12147	1702.5	1688.9	1675.1	1661.0
	0.17142	1702.8	1689.1	1675.0	1660.8
	0.19612	1703.0	1689.1	1675.0	1660.6
	0.22873	1703.1	1689.1	1674.9	1660.4
	0.26881	1703.2	1689.1	1674.7	1660.1
x_W = 0.700	0.00000	1689.2	1672.0	1654.8	1637.6
	0.02926	1688.6	1671.3	1654.1	1636.8
	0.05503	1687.9	1670.7	1653.4	1636.1
	0.07243	1687.5	1670.2	1652.8	1635.6
	0.12016	1686.4	1668.9	1651.5	1634.1
	0.17029	1685.1	1667.6	1650.1	1632.5

	0.19564	1684.4	1666.8	1649.3	1631.7
	0.23316	1683.4	1665.7	1648.1	1630.4
	0.26766	1682.4	1664.7	1647.0	1629.3
$x_W = 0.600$	0.00000	1655.4	1638.6	1621.3	1603.3
	0.02648	1654.5	1637.7	1620.3	1602.2
	0.04971	1653.7	1636.8	1619.3	1601.4
	0.07105	1652.8	1635.9	1618.5	1600.5
	0.12025	1650.9	1634.0	1616.6	1598.5
	0.17040	1649.0	1632.1	1614.6	1596.5
	0.19550	1648.0	1631.0	1613.5	1595.4
	0.23012	1646.5	1629.5	1612.1	1594.0
	0.26635	1645.0	1628.0	1610.5	1592.5
$x_W = 0.500$	0.00000	1618.4	1599.7	1581.0	1562.3
	0.02201	1617.7	1599.0	1580.2	1561.5
	0.05108	1616.6	1597.9	1579.1	1560.4
	0.07481	1615.8	1597.1	1578.3	1559.7
	0.12232	1614.1	1595.4	1576.6	1558.0
	0.17008	1612.4	1593.6	1574.8	1556.3
	0.19646	1611.4	1592.5	1573.9	1555.2
	0.23312	1610.0	1591.2	1572.5	1553.9
	0.27166	1608.5	1589.7	1571.0	1552.5
$x_W = 0.400$	0.00000	1581.7	1563.8	1545.7	1527.0
	0.02618	1581.0	1563.2	1544.9	1526.3
	0.05053	1580.3	1562.4	1544.3	1525.6
	0.07297	1579.6	1561.8	1543.6	1525.0
	0.12102	1578.2	1560.3	1542.1	1523.5
	0.17060	1576.7	1558.8	1540.7	1522.0
	0.19577	1576.0	1558.1	1539.9	1521.2
	0.23433	1574.7	1556.8	1538.7	1520.0
	0.27634	1573.4	1555.5	1537.3	1518.7
$x_W = 0.300$	0.00000	1549.7	1530.6	1511.6	1492.8
	0.02246	1549.3	1530.2	1511.2	1492.2
	0.05194	1548.5	1529.5	1510.4	1491.6

	0.07419	1548.1	1528.9	1509.9	1491.0
	0.12146	1546.9	1527.8	1508.7	1489.9
	0.16973	1545.7	1526.5	1507.5	1488.6
	0.19491	1545.1	1525.9	1506.8	1487.9
	0.23015	1544.1	1525.0	1505.9	1486.9
	0.27596	1542.9	1523.7	1504.6	1485.7
x_W = 0.200	0.00000	1522.4	1503.8	1484.9	1465.9
	0.02621	1522.0	1503.4	1484.5	1465.5
	0.04975	1521.7	1503.1	1484.2	1465.2
	0.07225	1521.3	1502.6	1483.8	1464.7
	0.12419	1520.4	1501.7	1482.9	1463.8
	0.16946	1519.5	1500.9	1482.0	1463.0
	0.19216	1519.1	1500.5	1481.6	1462.5
	0.23052	1518.3	1499.7	1480.8	1461.8
	0.27650	1517.4	1498.7	1479.8	1460.8
x_W = 0.100	0.00000	1497.6	1478.2	1458.9	1439.9
	0.02383	1497.4	1478.0	1458.7	1439.7
	0.05002	1497.2	1477.9	1458.6	1439.5
	0.07364	1497.1	1477.6	1458.5	1439.3
	0.12204	1496.6	1477.1	1457.8	1438.8
	0.17099	1496.0	1476.6	1457.3	1438.2
	0.19562	1495.7	1476.3	1457.0	1437.9
	0.23233	1495.2	1475.8	1456.5	1437.4
	0.27585	1494.5	1475.2	1455.8	1436.8
x_W = 0.000	0.00000	1477.2	1457.9	1438.6	1419.1
	0.02386	1477.2	1458.0	1438.6	1419.1
	0.05145	1477.2	1458.0	1438.6	1419.1
	0.07363	1477.2	1457.9	1438.6	1419.1
	0.12070	1477.1	1457.8	1438.4	1418.9
	0.17089	1476.8	1457.6	1438.2	1418.7
	0.19481	1476.7	1457.4	1438.1	1418.5
	0.23328	1476.4	1457.2	1437.8	1418.3
	0.27780	1476.0	1456.8	1437.4	1417.9

The uncertainty of the mole fraction x_W is equal to $\pm 1 \cdot 10^{-3}$. The uncertainty of molality is equal to $\pm 1.5 \cdot 10^{-5}$ mol·kg^{-1}.

Table S4. Speed of sound in the hexaglyme + DMF + W solution at pressure p = 0.100 ± 0.005MPa.

		$u/(\mathrm{m \cdot s^{-1}})$			
	m/(mol·kg^{-1})	293.15 K	298.15 K	303.15 K	308.15 K
x_W = 1.000	0.00000	1483.5	1496.8	1510.2	1520.7
	0.02654	1488.6	1501.5	1514.4	1524.5
	0.04718	1492.6	1505.1	1517.7	1527.5
	0.07348	1497.7	1509.7	1521.9	1531.3
	0.12072	1506.8	1518.0	1529.5	1538.1
	0.16577	1515.5	1525.9	1536.6	1544.7
	0.19290	1520.7	1530.7	1541.0	1548.6
	0.23377	1528.6	1537.9	1547.5	1554.5
	0.26703	1535.0	1543.7	1552.8	1559.3
x_W = 0.980	0.00000	1535.1	1543.9	1551.7	1557.4
	0.02630	1539.6	1548.0	1555.3	1560.7
	0.04688	1543.1	1551.2	1558.2	1563.2
	0.07142	1547.2	1555.0	1561.6	1566.3
	0.11931	1555.4	1562.4	1568.2	1572.2
	0.17341	1564.6	1570.8	1575.7	1579.0
	0.19399	1568.1	1574.0	1578.6	1581.5
	0.22823	1574.0	1579.3	1583.3	1585.8
	0.26482	1580.2	1585.0	1588.4	1590.4
x_W = 0.960	0.00000	1574.7	1579.0	1582.1	1584.1
	0.02806	1579.3	1583.1	1585.8	1587.4
	0.04993	1582.8	1586.2	1588.7	1590.0
	0.06868	1585.8	1588.9	1591.1	1592.2
	0.11870	1593.6	1595.8	1597.5	1597.8
	0.16749	1600.9	1602.3	1603.4	1603.2
	0.19471	1604.9	1605.9	1606.7	1606.1
	0.23961	1611.3	1611.6	1611.9	1610.8

	0.26936	1615.5	1615.3	1615.3	1613.8
$x_W = 0.940$	0.00000	1607.7	1608.1	1607.6	1606.2
	0.04917	1611.6	1611.7	1610.8	1609.0
	0.06764	1614.6	1614.3	1613.2	1611.1
	0.11687	1617.0	1616.6	1615.2	1612.8
	0.16487	1623.1	1622.1	1620.2	1617.2
	0.19164	1628.5	1627.0	1624.6	1621.0
	0.23578	1631.3	1629.5	1626.9	1622.9
	0.26501	1635.4	1633.3	1630.3	1625.9
	0.04917	1637.9	1635.6	1632.4	1627.7
$x_W = 0.920$	0.00000	1635.0	1632.1	1628.5	1624.3
	0.02492	1638.0	1634.7	1630.8	1626.3
	0.04866	1640.7	1637.2	1633.0	1628.2
	0.07143	1643.3	1639.5	1635.0	1629.9
	0.11787	1648.4	1644.0	1639.0	1633.4
	0.17385	1654.2	1649.2	1643.6	1637.4
	0.20086	1656.9	1651.6	1645.7	1639.2
	0.22911	1659.6	1654.0	1647.8	1641.1
	0.25937	1662.5	1656.5	1650.0	1643.0
$x_W = 0.900$	0.00000	1655.5	1649.8	1643.6	1636.9
	0.02702	1658.2	1652.2	1645.7	1638.8
	0.04911	1660.4	1654.1	1647.4	1640.2
	0.06985	1662.3	1655.8	1648.9	1641.5
	0.11608	1666.4	1659.3	1652.0	1644.2
	0.16915	1670.7	1663.0	1655.2	1647.0
	0.19264	1672.5	1664.5	1656.5	1648.2
	0.23327	1675.3	1666.9	1658.6	1649.9
	0.26404	1677.3	1668.6	1660.0	1651.2
$x_W = 0.800$	0.00000	1700.2	1686.2	1672.0	1657.7
	0.02631	1700.8	1686.6	1672.3	1657.9
	0.05015	1701.3	1687.0	1672.6	1658.1
	0.06965	1701.7	1687.3	1672.7	1658.2
	0.12002	1702.5	1687.8	1673.0	1658.3

	0.16883	1703.2	1688.2	1673.2	1658.3
	0.19503	1703.4	1688.4	1673.3	1658.2
	0.23372	1703.7	1688.5	1673.3	1658.1
	0.26945	1704.0	1688.6	1673.2	1657.9
$x_W = 0.700$	0.00000	1687.8	1670.7	1653.5	1636.3
	0.02721	1687.1	1669.9	1652.6	1635.4
	0.05019	1686.5	1669.2	1651.9	1634.6
	0.07042	1686.0	1668.7	1651.3	1634.1
	0.11807	1684.7	1667.3	1649.8	1632.5
	0.16552	1683.2	1665.6	1648.1	1630.7
	0.19639	1682.4	1664.8	1647.2	1629.8
	0.23262	1681.2	1663.5	1645.8	1628.4
	0.26930	1680.0	1662.2	1644.5	1627.0
$x_W = 0.600$	0.00000	1655.3	1637.0	1618.7	1600.4
	0.02582	1654.3	1636.0	1617.6	1599.4
	0.04807	1653.4	1635.1	1616.7	1598.4
	0.07046	1652.6	1634.2	1615.8	1597.6
	0.11933	1650.6	1632.2	1613.8	1595.5
	0.16988	1648.5	1630.0	1611.6	1593.3
	0.19224	1647.4	1628.9	1610.5	1592.2
	0.23002	1645.8	1627.4	1608.9	1590.6
	0.27268	1644.2	1625.7	1607.2	1588.9
$x_W = 0.500$	0.00000	1617.3	1598.6	1579.9	1561.3
	0.02620	1616.6	1597.9	1579.1	1560.5
	0.04908	1615.5	1596.8	1578.1	1559.4
	0.07135	1614.7	1596.0	1577.2	1558.5
	0.11924	1612.9	1594.2	1575.4	1556.7
	0.16660	1611.1	1592.3	1573.5	1554.8
	0.19558	1610.1	1591.2	1572.4	1553.8
	0.23001	1608.6	1589.7	1570.9	1552.3
	0.27069	1607.0	1588.1	1569.3	1550.7
$x_W = 0.400$	0.00000	1581.7	1562.8	1543.9	1525.1
	0.02533	1581.0	1562.1	1543.2	1524.4

	0.04807	1580.3	1561.4	1542.5	1523.7
	0.07303	1579.7	1560.7	1541.8	1523.1
	0.12068	1578.3	1559.3	1540.4	1521.7
	0.16770	1576.7	1557.8	1538.9	1520.1
	0.19931	1575.9	1557.0	1538.1	1519.4
	0.23383	1574.6	1555.7	1536.8	1518.1
	0.27474	1573.2	1554.3	1535.4	1516.7
$x_W = 0.300$	0.00000	1549.7	1530.6	1511.6	1492.8
	0.02667	1549.3	1530.3	1511.2	1492.4
	0.05097	1548.8	1529.7	1510.7	1491.9
	0.07071	1548.4	1529.3	1510.3	1491.4
	0.11946	1547.4	1528.4	1509.4	1490.5
	0.16816	1546.4	1527.4	1508.3	1489.5
	0.19414	1545.8	1526.9	1507.8	1488.9
	0.23318	1545.0	1526.0	1507.0	1488.1
	0.27451	1543.9	1524.8	1505.9	1487.0
$x_W = 0.200$	0.00000	1522.5	1503.3	1484.1	1465.1
	0.02406	1522.3	1503.1	1483.9	1464.9
	0.05074	1522.1	1502.8	1483.7	1464.6
	0.06912	1521.9	1502.6	1483.4	1464.4
	0.12024	1521.3	1502.0	1482.8	1463.8
	0.17291	1520.6	1501.4	1482.2	1463.2
	0.19690	1520.3	1501.1	1481.9	1462.9
	0.23388	1519.7	1500.5	1481.3	1462.3
	0.26538	1518.9	1499.7	1480.5	1461.5
$x_W = 0.100$	0.00000	1497.6	1478.2	1458.9	1439.9
	0.02556	1497.6	1478.2	1458.9	1439.9
	0.04951	1497.6	1478.1	1458.9	1439.8
	0.07371	1497.5	1478.1	1458.8	1439.7
	0.12028	1497.3	1477.9	1458.6	1439.5
	0.16628	1496.9	1477.5	1458.3	1439.2
	0.19362	1496.7	1477.3	1458.1	1439.0
	0.23091	1496.3	1477.0	1457.7	1438.7

	0.28006	1495.8	1476.5	1457.2	1438.2
x_W = 0.000	0.00000	1478.6	1459.2	1439.8	1420.5
	0.02672	1478.8	1459.4	1440.0	1420.7
	0.04932	1479.0	1459.5	1440.1	1420.8
	0.07293	1479.1	1459.6	1440.2	1420.9
	0.12086	1479.2	1459.8	1440.3	1421.0
	0.17102	1479.2	1459.7	1440.3	1421.0
	0.19284	1479.1	1459.7	1440.3	1421.0
	0.23187	1479.0	1459.5	1440.1	1420.8
	0.28013	1478.6	1459.2	1439.8	1420.5

The uncertainty of the mole fraction x_W is equal to $\pm1\cdot10^{-3}$. The uncertainty of molality is equal to $\pm1.5\cdot10^{-5}$ mol·kg^{-1}.

Table S5. The apparent molar volume of the pentaglyme in DMF + W mixture.

	$V_{\Phi,m}\cdot10^6$ / m$^3\cdot$mol^{-1}				
	m/mol·kg^{-1}	293.15 K	298.15 K	303.15 K	308.15 K
x_W = 1.000	0.02720	240.44	241.66	242.95	244.15
	0.04899	240.32	241.57	242.86	244.08
	0.07406	240.22	241.46	242.77	244.02
	0.12010	240.01	241.27	242.63	243.88
	0.17162	239.80	241.06	242.46	243.73
	0.19593	239.69	240.97	242.38	243.67
	0.23202	239.54	240.83	242.26	243.57
	0.26652	239.40	240.69	242.15	243.48
x_W = 0.980	0.02290	239.41	240.83	242.18	243.57
	0.05007	239.36	240.77	242.12	243.52
	0.07274	239.30	240.73	242.09	243.50
	0.12350	239.20	240.64	242.00	243.45
	0.17066	239.12	240.56	241.94	243.40
	0.19792	239.08	240.51	241.90	243.38
	0.22953	239.02	240.46	241.86	243.35
	0.26544	238.96	240.40	241.82	243.32
x_W = 0.960	0.02556	238.47	239.90	241.34	242.86

	0.05031	238.46	239.92	241.35	242.88
	0.07117	238.46	239.92	241.35	242.89
	0.12387	238.43	239.90	241.36	242.91
	0.17126	238.42	239.90	241.37	242.93
	0.19723	238.41	239.90	241.38	242.94
	0.23223	238.41	239.91	241.39	242.97
	0.26541	238.40	239.92	241.40	242.99
$x_W = 0.940$	0.02549	237.86	239.43	241.01	242.66
	0.04819	237.88	239.45	241.03	242.67
	0.07117	237.91	239.48	241.05	242.69
	0.12085	237.97	239.53	241.08	242.71
	0.17119	238.02	239.57	241.12	242.75
	0.19426	238.05	239.60	241.14	242.77
	0.23011	238.10	239.63	241.17	242.79
	0.26290	238.13	239.65	241.19	242.81
$x_W = 0.920$	0.02543	238.12	239.70	241.36	243.01
	0.04882	238.15	239.71	241.38	243.03
	0.07199	238.17	239.74	241.41	243.06
	0.12158	238.23	239.80	241.47	243.12
	0.17003	238.28	239.87	241.54	243.20
	0.19963	238.33	239.92	241.59	243.25
	0.23678	238.38	239.99	241.66	243.31
	0.26349	238.42	240.04	241.69	243.36
$x_W = 0.900$	0.02802	238.98	240.69	242.31	243.91
	0.05067	239.00	240.71	242.32	243.93
	0.06925	239.04	240.72	242.35	243.94
	0.12312	239.09	240.77	242.40	244.00
	0.16960	239.16	240.81	242.45	244.05
	0.19731	239.21	240.84	242.49	244.09
	0.23046	239.26	240.89	242.53	244.13
	0.26704	239.32	240.92	242.59	244.17
$x_W = 0.800$	0.02662	244.59	246.14	247.66	249.18
	0.05278	244.64	246.19	247.70	249.21

	0.07110	244.68	246.22	247.74	249.23
	0.12147	244.77	246.30	247.81	249.28
	0.17142	244.87	246.40	247.90	249.34
	0.19612	244.92	246.45	247.94	249.37
	0.22873	245.00	246.52	248.00	249.42
	0.26881	245.09	246.61	248.08	249.46
xw = 0.700	0.02926	249.78	251.12	252.47	253.86
	0.05503	249.84	251.19	252.53	253.91
	0.07243	249.89	251.22	252.58	253.95
	0.12016	250.00	251.34	252.68	254.05
	0.17029	250.14	251.48	252.81	254.18
	0.19564	250.21	251.55	252.88	254.24
	0.23316	250.32	251.66	252.98	254.33
	0.26766	250.42	251.76	253.08	254.43
xw = 0.600	0.02648	253.37	254.71	256.00	257.39
	0.04971	253.43	254.76	256.06	257.44
	0.07105	253.49	254.81	256.11	257.48
	0.12025	253.62	254.94	256.23	257.60
	0.17040	253.75	255.08	256.37	257.72
	0.19550	253.82	255.14	256.43	257.78
	0.23012	253.92	255.23	256.52	257.86
	0.26635	254.02	255.32	256.61	257.94
xw = 0.500	0.02201	255.78	257.10	258.44	259.81
	0.05108	255.82	257.15	258.48	259.85
	0.07481	255.85	257.20	258.51	259.88
	0.12232	255.93	257.26	258.59	259.95
	0.17008	256.02	257.34	258.68	260.02
	0.19646	256.07	257.39	258.73	260.07
	0.23312	256.15	257.46	258.79	260.12
	0.27166	256.22	257.53	258.85	260.18
xw = 0.400	0.02618	257.08	258.46	259.82	261.14
	0.05053	257.12	258.50	259.87	261.18
	0.07297	257.17	258.54	259.90	261.22

	0.12102	257.25	258.61	259.97	261.30
	0.17060	257.35	258.69	260.04	261.39
	0.19577	257.40	258.73	260.08	261.44
	0.23433	257.48	258.80	260.16	261.51
	0.27634	257.56	258.87	260.22	261.58
xw = 0.300	0.02246	257.30	258.57	259.92	261.27
	0.05194	257.34	258.62	259.96	261.31
	0.07419	257.37	258.65	259.99	261.35
	0.12146	257.45	258.73	260.06	261.40
	0.16973	257.53	258.82	260.14	261.47
	0.19491	257.58	258.87	260.19	261.51
	0.23015	257.65	258.94	260.25	261.57
	0.27596	257.73	259.02	260.32	261.64
xw = 0.200	0.02621	255.60	256.96	258.30	259.58
	0.04975	255.67	257.01	258.35	259.62
	0.07225	255.71	257.05	258.38	259.66
	0.12419	255.84	257.16	258.48	259.75
	0.16946	255.95	257.27	258.56	259.85
	0.19216	256.02	257.32	258.60	259.88
	0.23052	256.12	257.41	258.68	259.95
	0.27650	256.22	257.51	258.76	260.05
xw = 0.100	0.02383	252.89	254.21	255.48	256.80
	0.05002	253.04	254.34	255.62	256.94
	0.07364	253.14	254.46	255.75	257.06
	0.12204	253.42	254.73	256.02	257.32
	0.17099	253.71	255.01	256.29	257.60
	0.19562	253.86	255.16	256.44	257.75
	0.23233	254.09	255.38	256.65	257.95
	0.27585	254.33	255.66	256.91	258.17
xw = 0.000	0.02386	250.47	251.68	252.89	253.98
	0.05145	250.77	252.02	253.21	254.31
	0.07363	251.04	252.27	253.46	254.55
	0.12070	251.63	252.83	253.99	255.12

0.17089	252.28	253.45	254.59	255.73
0.19481	252.59	253.74	254.88	256.03
0.23328	253.10	254.24	255.35	256.51
0.27780	253.72	254.82	255.92	257.10

The uncertainty of the mole fraction x_W is equal to $\pm 1\cdot 10^{-3}$. The uncertainty of molality is equal to $\pm 1.5\cdot 10^{-5}$ mol·kg^{-1}.

Table S6. The apparent molar volume of the hexaglyme in DMF + W mixture.

	$V_{\Phi,m}\cdot 10^6$ / m$^3\cdot$mol^{-1}				
	m/mol·kg^{-1}	293.15 K	298.15 K	303.15 K	308.15 K
x_W = 1.000	0.02654	279.12	280.58	282.05	283.54
	0.04718	279.00	280.45	281.98	283.47
	0.07348	278.88	280.31	281.90	283.38
	0.12072	278.65	280.11	281.69	283.19
	0.16577	278.43	279.93	281.49	283.03
	0.19290	278.30	279.83	281.41	282.93
	0.23377	278.11	279.65	281.25	282.79
	0.26703	277.94	279.50	281.12	282.68
x_W = 0.980	0.02630	276.37	277.93	279.48	281.01
	0.04688	276.31	277.89	279.42	280.99
	0.07142	276.24	277.84	279.37	280.94
	0.11931	276.11	277.74	279.28	280.86
	0.17341	275.98	277.63	279.20	280.78
	0.19399	275.93	277.59	279.18	280.75
	0.22823	275.86	277.51	279.09	280.70
	0.26482	275.79	277.43	279.02	280.66
x_W = 0.960	0.02806	275.29	277.05	278.80	280.47
	0.04993	275.25	277.01	278.78	280.45
	0.06868	275.24	276.99	278.77	280.44
	0.11870	275.19	276.95	278.73	280.40
	0.16749	275.16	276.92	278.68	280.35
	0.19471	275.15	276.89	278.66	280.33
	0.23961	275.08	276.85	278.63	280.31

	0.26936	275.05	276.82	278.60	280.28
$x_W = 0.940$	0.02764	274.43	276.25	278.13	279.94
	0.04917	274.44	276.26	278.15	279.95
	0.06764	274.46	276.28	278.17	279.96
	0.11687	274.48	276.31	278.19	279.98
	0.16487	274.51	276.34	278.23	280.00
	0.19164	274.53	276.36	278.25	280.01
	0.23578	274.58	276.40	278.28	280.04
	0.26501	274.61	276.41	278.31	280.06
$x_W = 0.920$	0.02492	274.92	276.84	278.63	280.45
	0.04866	274.95	276.87	278.66	280.48
	0.07143	274.99	276.90	278.68	280.51
	0.11787	275.05	276.95	278.73	280.54
	0.17385	275.14	277.00	278.80	280.60
	0.20086	275.18	277.04	278.83	280.63
	0.22911	275.24	277.09	278.87	280.67
	0.25937	275.28	277.14	278.91	280.70
$x_W = 0.900$	0.02702	275.58	277.39	279.23	281.00
	0.04911	275.61	277.42	279.25	281.02
	0.06985	275.61	277.44	279.27	281.04
	0.11608	275.66	277.49	279.32	281.09
	0.16915	275.72	277.55	279.38	281.14
	0.19264	275.75	277.58	279.41	281.17
	0.23327	275.82	277.64	279.46	281.22
	0.26404	275.87	277.67	279.50	281.25
$x_W = 0.800$	0.02631	281.50	283.10	284.72	286.51
	0.05015	281.55	283.14	284.77	286.57
	0.06965	281.63	283.20	284.81	286.62
	0.12002	281.77	283.33	284.93	286.74
	0.16883	281.89	283.45	285.03	286.85
	0.19503	281.95	283.52	285.09	286.90
	0.23372	282.05	283.62	285.19	287.00
	0.26945	282.15	283.71	285.28	287.10

$x_W = 0.700$	0.02721	287.66	289.22	290.82	292.37
	0.05019	287.69	289.26	290.86	292.40
	0.07042	287.73	289.30	290.89	292.44
	0.11807	287.82	289.38	290.95	292.52
	0.16552	287.91	289.46	291.03	292.60
	0.19639	287.98	289.52	291.08	292.66
	0.23262	288.06	289.59	291.15	292.72
	0.26930	288.12	289.65	291.20	292.78
$x_W = 0.600$	0.02582	291.83	293.30	294.79	296.29
	0.04807	291.87	293.34	294.83	296.34
	0.07046	291.91	293.38	294.87	296.37
	0.11933	291.99	293.45	294.94	296.45
	0.16988	292.08	293.54	295.03	296.53
	0.19224	292.13	293.58	295.07	296.57
	0.23002	292.20	293.66	295.14	296.63
	0.27268	292.28	293.73	295.21	296.70
$x_W = 0.500$	0.02620	294.78	296.26	297.77	299.30
	0.04908	294.81	296.29	297.81	299.33
	0.07135	294.84	296.32	297.83	299.35
	0.11924	294.90	296.38	297.89	299.42
	0.16660	294.97	296.45	297.96	299.48
	0.19558	295.02	296.50	298.01	299.53
	0.23001	295.08	296.55	298.06	299.58
	0.27069	295.15	296.61	298.11	299.64
$x_W = 0.400$	0.02533	295.86	297.41	298.86	300.41
	0.04807	295.88	297.43	298.89	300.44
	0.07303	295.91	297.45	298.92	300.47
	0.12068	295.96	297.49	298.96	300.51
	0.16770	296.03	297.54	299.02	300.57
	0.19931	296.07	297.58	299.07	300.61
	0.23383	296.11	297.61	299.11	300.65
	0.27474	296.16	297.65	299.16	300.69
$x_W = 0.300$	0.02667	295.54	297.03	298.58	300.12

	0.05097	295.58	297.07	298.62	300.15
	0.07071	295.61	297.10	298.65	300.19
	0.11946	295.70	297.19	298.73	300.25
	0.16816	295.77	297.28	298.80	300.32
	0.19414	295.83	297.33	298.84	300.36
	0.23318	295.91	297.41	298.91	300.43
	0.27451	295.98	297.47	298.98	300.49
x_W = 0.200	0.02406	293.33	294.78	296.33	297.88
	0.05074	293.39	294.85	296.38	297.93
	0.06912	293.43	294.90	296.41	297.96
	0.12024	293.55	295.00	296.49	298.04
	0.17291	293.68	295.14	296.59	298.14
	0.19690	293.75	295.20	296.64	298.18
	0.23388	293.85	295.29	296.73	298.26
	0.26538	293.92	295.36	296.80	298.31
x_W = 0.100	0.02556	290.71	292.10	293.71	295.16
	0.04951	290.86	292.30	293.85	295.30
	0.07371	291.01	292.48	293.99	295.47
	0.12028	291.32	292.82	294.32	295.77
	0.16628	291.65	293.19	294.65	296.09
	0.19362	291.85	293.41	294.86	296.29
	0.23091	292.14	293.73	295.15	296.57
	0.28006	292.54	294.16	295.55	296.96
x_W = 0.000	0.02672	287.74	289.14	290.58	291.96
	0.04932	288.06	289.45	290.90	292.27
	0.07293	288.43	289.79	291.24	292.58
	0.12086	289.19	290.49	291.99	293.26
	0.17102	290.01	291.26	292.80	293.99
	0.19284	290.37	291.60	293.16	294.32
	0.23187	291.05	292.23	293.82	294.92
	0.28013	291.91	293.04	294.67	295.69

The uncertainty of the mole fraction x_W is equal to $\pm 1\cdot 10^{-3}$. The uncertainty of molality is equal to $\pm 1.5\cdot 10^{-5}$ mol·kg^{-1}.

Table S7. Isentropic compressibility coefficient of the pentaglyme + DMF + W solution.

	$\kappa_S \cdot 10^{10}$ / Pa^{-1}				
	m/(mol·kg^{-1})	293.15 K	298.15 K	303.15 K	308.15 K
x_W = 1.000	0.00000	4.55450	4.47369	4.40664	4.35249
	0.02720	4.52376	4.44623	4.38222	4.33032
	0.04899	4.49939	4.42433	4.36259	4.31282
	0.07406	4.47124	4.39990	4.34046	4.29286
	0.12010	4.42030	4.35493	4.30053	4.25654
	0.17162	4.36439	4.30554	4.25615	4.21634
	0.19593	4.33813	4.28215	4.23603	4.19714
	0.23202	4.30006	4.24829	4.20560	4.16944
	0.26652	4.26433	4.21663	4.17673	4.14323
x_W = 0.980	0.00000	4.25744	4.21482	4.18188	4.15824
	0.02290	4.23598	4.19541	4.16494	4.14259
	0.05007	4.21148	4.17299	4.14447	4.12451
	0.07274	4.19041	4.15446	4.12781	4.10964
	0.12350	4.14498	4.11247	4.09008	4.07601
	0.17066	4.10335	4.07464	4.05586	4.04525
	0.19792	4.07952	4.05325	4.03677	4.02790
	0.22953	4.05185	4.02802	4.01438	4.00775
	0.26544	4.02149	4.00010	3.98878	3.98489
x_W = 0.960	0.00000	4.03855	4.02365	4.01620	4.01456
	0.02556	4.01726	4.00461	3.99833	3.99884
	0.05031	3.99675	3.98633	3.98170	3.98382
	0.07117	3.98036	3.97109	3.96807	3.97127
	0.12387	3.93982	3.93437	3.93457	3.94142
	0.17126	3.90491	3.90267	3.90551	3.91543
	0.19723	3.88670	3.88604	3.89043	3.90186
	0.23223	3.86293	3.86388	3.87078	3.88374
	0.26541	3.84055	3.84402	3.85245	3.86739
x_W = 0.940	0.00000	3.86323	3.87182	3.88401	3.90072
	0.02549	3.84559	3.85519	3.86935	3.88802
	0.04819	3.83029	3.84089	3.85699	3.87664

	0.07117	3.81508	3.82666	3.84420	3.86529
	0.12085	3.78369	3.79674	3.81806	3.84154
	0.17119	3.75318	3.76814	3.79269	3.81899
	0.19426	3.73945	3.75534	3.78172	3.80848
	0.23011	3.71915	3.73643	3.76506	3.79367
	0.26290	3.70116	3.71936	3.75020	3.78018
$x_W = 0.920$	0.00000	3.74189	3.76484	3.79172	3.82218
	0.02543	3.72734	3.75165	3.77990	3.81127
	0.04882	3.71448	3.73968	3.76928	3.80156
	0.07199	3.70218	3.72826	3.75921	3.79194
	0.12158	3.67707	3.70536	3.73852	3.77305
	0.17003	3.65420	3.68467	3.72003	3.75589
	0.19963	3.64085	3.67262	3.70973	3.74649
	0.23678	3.62562	3.65868	3.69708	3.73472
	0.26349	3.61495	3.64886	3.68856	3.72708
$x_W = 0.900$	0.00000	3.64354	3.67921	3.71855	3.76068
	0.02802	3.63068	3.66763	3.70779	3.75123
	0.05067	3.62061	3.65841	3.69940	3.74369
	0.06925	3.61231	3.65094	3.69278	3.73748
	0.12312	3.58910	3.62980	3.67371	3.72054
	0.16960	3.57033	3.61265	3.65818	3.70625
	0.19731	3.55937	3.60291	3.64924	3.69814
	0.23046	3.54676	3.59107	3.63861	3.68876
	0.26704	3.53314	3.57906	3.62737	3.67876
$x_W = 0.800$	0.00000	3.45677	3.52380	3.59447	3.66868
	0.02662	3.45315	3.52074	3.59191	3.66651
	0.05278	3.44993	3.51797	3.58954	3.66458
	0.07110	3.44772	3.51607	3.58798	3.66328
	0.12147	3.44207	3.51138	3.58409	3.66017
	0.17142	3.43714	3.50731	3.58086	3.65764
	0.19612	3.43490	3.50552	3.57946	3.65659
	0.22873	3.43224	3.50343	3.57788	3.65545
	0.26881	3.42928	3.50118	3.57623	3.65434

$x_W = 0.700$	0.00000	3.52061	3.60912	3.70067	3.79601
	0.02926	3.52118	3.61032	3.70226	3.79799
	0.05503	3.52229	3.61125	3.70376	3.79981
	0.07243	3.52293	3.61236	3.70530	3.80097
	0.12016	3.52505	3.61498	3.70807	3.80498
	0.17029	3.52753	3.61814	3.71187	3.80945
	0.19564	3.52901	3.61993	3.71398	3.81190
	0.23316	3.53141	3.62276	3.71727	3.81565
	0.26766	3.53385	3.62561	3.72053	3.81935
$x_W = 0.600$	0.00000	3.69117	3.78475	3.88439	3.99115
	0.02648	3.69397	3.78777	3.88765	3.99462
	0.04971	3.69614	3.79041	3.89090	3.99752
	0.07105	3.69888	3.79314	3.89336	4.00061
	0.12025	3.70463	3.79934	3.89998	4.00759
	0.17040	3.71091	3.80566	3.90707	4.01501
	0.19550	3.71401	3.80959	3.91078	4.01914
	0.23012	3.71885	3.81458	3.91604	4.02429
	0.26635	3.72398	3.81998	3.92171	4.03017
$x_W = 0.500$	0.00000	3.89235	4.00296	4.11824	4.23779
	0.02201	3.89462	4.00541	4.12086	4.24049
	0.05108	3.89795	4.00906	4.12464	4.24462
	0.07481	3.90037	4.01160	4.12732	4.24722
	0.12232	3.90593	4.01744	4.13349	4.25359
	0.17008	3.91178	4.02367	4.14009	4.26024
	0.19646	3.91536	4.02758	4.14366	4.26438
	0.23312	3.92002	4.03235	4.14898	4.26952
	0.27166	3.92537	4.03796	4.15480	4.27540
$x_W = 0.400$	0.00000	4.10797	4.22273	4.34387	4.47262
	0.02618	4.11004	4.22448	4.34622	4.47516
	0.05053	4.11220	4.22709	4.34818	4.47757
	0.07297	4.11416	4.22920	4.35074	4.47944
	0.12102	4.11836	4.23379	4.35562	4.48499
	0.17060	4.12322	4.23894	4.36081	4.49046

	0.19577	4.12583	4.24151	4.36386	4.49360
	0.23433	4.13014	4.24618	4.36836	4.49833
	0.27634	4.13481	4.25100	4.37341	4.50366
x_W = 0.300	0.00000	4.31177	4.44153	4.57658	4.71634
	0.02246	4.31250	4.44249	4.57749	4.71783
	0.05194	4.31429	4.44439	4.57975	4.71978
	0.07419	4.31538	4.44571	4.58128	4.72160
	0.12146	4.31844	4.44884	4.58471	4.72491
	0.16973	4.32177	4.45277	4.58862	4.72961
	0.19491	4.32358	4.45451	4.59077	4.73193
	0.23015	4.32655	4.45753	4.59419	4.73541
	0.27596	4.33055	4.46180	4.59851	4.74019
x_W = 0.200	0.00000	4.49783	4.63246	4.77458	4.92398
	0.02621	4.49766	4.63239	4.77461	4.92411
	0.04975	4.49737	4.63218	4.77456	4.92415
	0.07225	4.49768	4.63266	4.77509	4.92466
	0.12419	4.49828	4.63347	4.77606	4.92574
	0.16946	4.49932	4.63442	4.77723	4.92728
	0.19216	4.49986	4.63543	4.77824	4.92824
	0.23052	4.50148	4.63697	4.77987	4.92999
	0.27650	4.50368	4.63930	4.78226	4.93248
x_W = 0.100	0.00000	4.67487	4.82249	4.97583	5.13428
	0.02383	4.67315	4.82074	4.97404	5.13274
	0.05002	4.67141	4.81883	4.97209	5.13083
	0.07364	4.66983	4.81762	4.97072	5.12945
	0.12204	4.66787	4.81538	4.96866	5.12717
	0.17099	4.66641	4.81385	4.96714	5.12559
	0.19562	4.66591	4.81324	4.96659	5.12521
	0.23233	4.66565	4.81307	4.96626	5.12478
	0.27585	4.66586	4.81295	4.96639	5.12484
x_W = 0.000	0.00000	4.83034	4.98376	5.14480	5.31415
	0.02386	4.82692	4.98022	5.14115	5.31042
	0.05145	4.82325	4.97643	5.13729	5.30648

0.07363	4.82052	4.97368	5.13444	5.30352
0.12070	4.81560	4.96838	5.12916	5.29798
0.17089	4.81151	4.96415	5.12462	5.29344
0.19481	4.80978	4.96242	5.12276	5.29166
0.23328	4.80782	4.96024	5.12055	5.28932
0.27780	4.80621	4.95862	5.11871	5.28752

The uncertainty of the mole fraction x_W is equal to $\pm 1\cdot 10^{-3}$. The uncertainty of molality is equal to $\pm 1.5\cdot 10^{-5}$ mol·kg^{-1}.

Table S8. Isentropic compressibility coefficient of the hexaglyme + DMF + W solution.

		$\kappa_S \cdot 10^{10}$ / Pa^{-1}			
	m/(mol·kg^{-1})	293.15 K	298.15 K	303.15 K	308.15 K
x_W = 1.000	0.00000	4.55203	4.47667	4.40375	4.35018
	0.02654	4.51711	4.44509	4.37595	4.32527
	0.04718	4.49002	4.42109	4.35433	4.30580
	0.07348	4.45585	4.39073	4.32704	4.28132
	0.12072	4.39579	4.33675	4.27836	4.23803
	0.16577	4.33951	4.28631	4.23353	4.19676
	0.19290	4.30636	4.25613	4.20619	4.17260
	0.23377	4.25676	4.21145	4.16621	4.13650
	0.26703	4.21719	4.17593	4.13405	4.10749
x_W = 0.980	0.00000	4.25300	4.21103	4.17595	4.15342
	0.02630	4.22435	4.18510	4.15312	4.13248
	0.04688	4.20226	4.16503	4.13497	4.11666
	0.07142	4.17657	4.14139	4.11380	4.09731
	0.11931	4.12608	4.09597	4.07319	4.06078
	0.17341	4.07054	4.04542	4.02791	4.01951
	0.19399	4.04973	4.02644	4.01068	4.00444
	0.22823	4.01508	3.99530	3.98291	3.97888
	0.26482	3.97911	3.96229	3.95321	3.95187
x_W = 0.960	0.00000	4.04059	4.02622	4.01874	4.01761
	0.02806	4.01310	4.00156	3.99632	3.99734
	0.04993	3.99233	3.98300	3.97891	3.98154

	0.06868	3.97466	3.96699	3.96453	3.96822
	0.11870	3.92918	3.92633	3.92661	3.93442
	0.16749	3.88716	3.88849	3.89191	3.90227
	0.19471	3.86441	3.86779	3.87275	3.88510
	0.23961	3.82832	3.83520	3.84268	3.85748
	0.26936	3.80492	3.81426	3.82322	3.83994
x_W = 0.940	0.00000	3.87428	3.88098	3.89273	3.90948
	0.02764	3.85171	3.85995	3.87368	3.89239
	0.04917	3.83448	3.84469	3.85941	3.87957
	0.06764	3.82063	3.83136	3.84753	3.86913
	0.11687	3.78555	3.79920	3.81779	3.84222
	0.16487	3.75447	3.77051	3.79146	3.81869
	0.19164	3.73833	3.75578	3.77767	3.80675
	0.23578	3.71438	3.73324	3.75700	3.78789
	0.26501	3.69968	3.71948	3.74416	3.77640
x_W = 0.920	0.00000	3.74371	3.76665	3.79357	3.82407
	0.02492	3.72672	3.75148	3.77975	3.81162
	0.04866	3.71139	3.73705	3.76665	3.79987
	0.07143	3.69676	3.72375	3.75469	3.78926
	0.11787	3.66820	3.69781	3.73092	3.76769
	0.17385	3.63592	3.66808	3.70375	3.74310
	0.20086	3.62099	3.65441	3.69135	3.73199
	0.22911	3.60606	3.64073	3.67892	3.72039
	0.25937	3.59020	3.62652	3.66596	3.70868
x_W = 0.900	0.00000	3.64969	3.68545	3.72441	3.76664
	0.02702	3.63441	3.67142	3.71164	3.75470
	0.04911	3.62206	3.66032	3.70136	3.74570
	0.06985	3.61126	3.65034	3.69220	3.73738
	0.11608	3.58805	3.62959	3.67310	3.71993
	0.16915	3.56357	3.60754	3.65309	3.70156
	0.19264	3.55331	3.59849	3.64485	3.69370
	0.23327	3.53708	3.58384	3.63141	3.68192
	0.26404	3.52543	3.57337	3.62217	3.67306

x_W = 0.800	0.00000	3.45983	3.53135	3.60581	3.68304
	0.02631	3.45491	3.52699	3.60194	3.67958
	0.05015	3.45066	3.52320	3.59858	3.67668
	0.06965	3.44730	3.52025	3.59602	3.67444
	0.12002	3.43927	3.51318	3.58984	3.66915
	0.16883	3.43230	3.50714	3.58466	3.66476
	0.19503	3.42890	3.50420	3.58220	3.66273
	0.23372	3.42427	3.50029	3.57889	3.66010
	0.26945	3.42042	3.49711	3.57628	3.65804
x_W = 0.700	0.00000	3.52613	3.61464	3.70669	3.80213
	0.02721	3.52656	3.61544	3.70785	3.80357
	0.05019	3.52704	3.61630	3.70896	3.80490
	0.07042	3.52762	3.61715	3.71006	3.80616
	0.11807	3.52933	3.61953	3.71302	3.80960
	0.16552	3.53157	3.62245	3.71654	3.81356
	0.19639	3.53331	3.62470	3.71914	3.81646
	0.23262	3.53570	3.62761	3.72249	3.82015
	0.26930	3.53843	3.63089	3.72622	3.82422
x_W = 0.600	0.00000	3.69195	3.79236	3.89690	4.00522
	0.02582	3.69421	3.79489	3.89961	4.00811
	0.04807	3.69625	3.79713	3.90206	4.01080
	0.07046	3.69845	3.79954	3.90469	4.01356
	0.11933	3.70358	3.80512	3.91070	4.01998
	0.16988	3.70947	3.81148	3.91748	4.02719
	0.19224	3.71225	3.81444	3.92063	4.03054
	0.23002	3.71721	3.81973	3.92627	4.03646
	0.27268	3.72317	3.82611	3.93300	4.04355
x_W = 0.500	0.00000	3.89770	4.00833	4.12362	4.24326
	0.02620	3.89988	4.01074	4.12627	4.24611
	0.04908	3.90195	4.01297	4.12867	4.24869
	0.07135	3.90399	4.01527	4.13115	4.25131
	0.11924	3.90884	4.02053	4.13679	4.25725
	0.16660	3.91407	4.02618	4.14283	4.26360

	0.19558	3.91750	4.02993	4.14677	4.26774
	0.23001	3.92181	4.03456	4.15169	4.27284
	0.27069	3.92725	4.04036	4.15783	4.27917
x_W = 0.400	0.00000	4.10802	4.22852	4.35384	4.48370
	0.02533	4.10928	4.22988	4.35528	4.48530
	0.04807	4.11050	4.23119	4.35673	4.48685
	0.07303	4.11203	4.23282	4.35846	4.48869
	0.12068	4.11524	4.23623	4.36207	4.49252
	0.16770	4.11892	4.24006	4.36613	4.49675
	0.19931	4.12161	4.24291	4.36908	4.49987
	0.23383	4.12483	4.24624	4.37260	4.50346
	0.27474	4.12901	4.25057	4.37706	4.50809
x_W = 0.300	0.00000	4.31179	4.44154	4.57658	4.71636
	0.02667	4.31150	4.44128	4.57637	4.71617
	0.05097	4.31134	4.44122	4.57634	4.71625
	0.07071	4.31137	4.44127	4.57643	4.71637
	0.11946	4.31180	4.44180	4.57705	4.71703
	0.16816	4.31292	4.44292	4.57822	4.71832
	0.19414	4.31370	4.44377	4.57907	4.71925
	0.23318	4.31529	4.44536	4.58072	4.72095
	0.27451	4.31735	4.44745	4.58287	4.72319
x_W = 0.200	0.00000	4.49688	4.63554	4.77997	4.92962
	0.02406	4.49495	4.63363	4.77803	4.92764
	0.05074	4.49298	4.63168	4.77604	4.92568
	0.06912	4.49180	4.63043	4.77483	4.92452
	0.12024	4.48907	4.62765	4.77195	4.92167
	0.17291	4.48711	4.62562	4.76989	4.91962
	0.19690	4.48651	4.62497	4.76923	4.91897
	0.23388	4.48602	4.62439	4.76859	4.91830
	0.26538	4.48594	4.62422	4.76837	4.91811
x_W = 0.100	0.00000	4.67488	4.82254	4.97583	5.13428
	0.02556	4.67104	4.81861	4.97179	5.13012
	0.04951	4.66772	4.81517	4.96825	5.12646

	0.07371	4.66463	4.81198	4.96496	5.12307
	0.12028	4.65947	4.80651	4.95933	5.11727
	0.16628	4.65531	4.80218	4.95473	5.11253
	0.19362	4.65329	4.79999	4.95248	5.11014
	0.23091	4.65104	4.79764	4.94992	5.10751
	0.28006	4.64905	4.79547	4.94754	5.10493
x_W = 0.000	0.00000	4.82088	4.97517	5.13627	5.30367
	0.02672	4.81486	4.96895	5.12980	5.29697
	0.04932	4.81005	4.96403	5.12468	5.29176
	0.07293	4.80539	4.95921	5.11972	5.28663
	0.12086	4.79702	4.95053	5.11070	5.27727
	0.17102	4.78973	4.94295	5.10278	5.26909
	0.19284	4.78704	4.94015	5.09987	5.26606
	0.23187	4.78303	4.93582	5.09542	5.26130
	0.28013	4.77927	4.93180	5.09116	5.25679

The uncertainty of the mole fraction x_W is equal to $\pm 1\cdot 10^{-3}$. The uncertainty of molality is equal to $\pm 1.5\cdot 10^{-5}$ mol·kg^{-1}.

Table S9. The apparent molar isentropic compression of pentaglyme in the DMF + W solvent.

		$K_{S,\Phi,m}\cdot 10^{14}/\left(m^3\cdot Pa^{-1}\cdot mol^{-1}\right)$			
	m/(mol·kg^{-1})	293.15 K	298.15 K	303.15 K	308.15 K
x_W = 1.000	0.02720	–0.44	0.62	1.62	2.37
	0.04899	–0.45	0.58	1.56	2.38
	0.07406	–0.52	0.63	1.56	2.38
	0.12010	–0.58	0.59	1.56	2.34
	0.17162	–0.63	0.55	1.51	2.30
	0.19593	–0.66	0.51	1.52	2.25
	0.23202	–0.69	0.49	1.48	2.22
	0.26652	–0.70	0.48	1.45	2.19
x_W = 0.980	0.02290	0.75	1.60	2.65	3.21
	0.05007	0.88	1.66	2.52	3.26
	0.07274	0.79	1.67	2.52	3.28

	0.12350	0.79	1.58	2.42	3.22
	0.17066	0.76	1.56	2.39	3.18
	0.19792	0.74	1.55	2.39	3.17
	0.22953	0.71	1.52	2.37	3.15
	0.26544	0.70	1.50	2.33	3.12
x_W = 0.960	0.02556	1.24	2.13	2.62	3.51
	0.05031	1.21	2.12	2.71	3.51
	0.07117	1.30	2.11	2.77	3.51
	0.12387	1.41	2.20	2.87	3.62
	0.17126	1.49	2.27	2.92	3.68
	0.19723	1.55	2.32	2.98	3.72
	0.23223	1.63	2.36	3.05	3.76
	0.26541	1.68	2.43	3.09	3.81
x_W = 0.940	0.02549	2.22	2.68	3.54	4.41
	0.04819	2.27	2.75	3.66	4.37
	0.07117	2.30	2.79	3.64	4.36
	0.12085	2.41	2.86	3.71	4.38
	0.17119	2.50	2.95	3.78	4.46
	0.19426	2.52	2.98	3.82	4.46
	0.23011	2.59	3.05	3.88	4.52
	0.26290	2.64	3.09	3.92	4.56
x_W = 0.920	0.02543	3.15	3.79	4.44	4.93
	0.04882	3.23	3.79	4.47	4.98
	0.07199	3.30	3.84	4.53	4.98
	0.12158	3.42	3.98	4.62	5.10
	0.17003	3.55	4.11	4.74	5.20
	0.19963	3.61	4.18	4.83	5.29
	0.23678	3.73	4.28	4.91	5.36
	0.26349	3.80	4.34	4.97	5.43
x_W = 0.900	0.02802	4.08	4.69	5.12	5.75
	0.05067	4.13	4.69	5.16	5.75
	0.06925	4.12	4.70	5.21	5.74
	0.12312	4.16	4.72	5.24	5.79

	0.16960	4.22	4.77	5.29	5.81
	0.19731	4.25	4.80	5.32	5.83
	0.23046	4.29	4.82	5.34	5.86
	0.26704	4.32	4.86	5.36	5.89
$x_W = 0.800$	0.02662	7.09	7.45	7.99	8.39
	0.05278	7.16	7.53	7.91	8.31
	0.07110	7.19	7.57	7.98	8.33
	0.12147	7.20	7.62	8.02	8.40
	0.17142	7.28	7.67	8.07	8.46
	0.19612	7.30	7.70	8.11	8.51
	0.22873	7.34	7.74	8.14	8.54
	0.26881	7.39	7.80	8.19	8.58
$x_W = 0.700$	0.02926	9.03	9.48	9.95	10.28
	0.05503	9.10	9.48	9.96	10.38
	0.07243	9.12	9.54	9.92	10.38
	0.12016	9.19	9.57	10.00	10.42
	0.17029	9.22	9.63	10.06	10.48
	0.19564	9.26	9.65	10.07	10.51
	0.23316	9.31	9.72	10.12	10.57
	0.26766	9.35	9.75	10.16	10.61
$x_W = 0.600$	0.02648	10.48	10.83	11.19	11.59
	0.04971	10.43	10.86	11.23	11.63
	0.07105	10.46	10.87	11.23	11.70
	0.12025	10.53	10.91	11.32	11.72
	0.17040	10.59	10.97	11.37	11.77
	0.19550	10.62	11.02	11.42	11.82
	0.23012	10.66	11.05	11.45	11.85
	0.26635	10.70	11.09	11.48	11.89
$x_W = 0.500$	0.02201	11.12	11.52	11.94	12.38
	0.05108	11.08	11.48	11.90	12.34
	0.07481	11.07	11.47	11.89	12.33
	0.12232	11.12	11.52	11.94	12.38
	0.17008	11.19	11.59	12.01	12.42

	0.19646	11.19	11.62	12.05	12.46
	0.23312	11.24	11.66	12.09	12.51
	0.27166	11.28	11.71	12.14	12.54
x_W = 0.400	0.02618	11.31	11.72	12.14	12.58
	0.05053	11.38	11.78	12.21	12.78
	0.07297	11.45	11.86	12.29	12.74
	0.12102	11.49	11.89	12.32	12.78
	0.17060	11.54	11.94	12.37	12.83
	0.19577	11.57	11.98	12.41	12.87
	0.23433	11.61	12.02	12.45	12.91
	0.27634	11.64	12.05	12.49	12.95
x_W = 0.300	0.02246	11.59	12.01	12.47	12.94
	0.05194	11.65	12.08	12.53	13.00
	0.07419	11.65	12.08	12.53	13.09
	0.12146	11.68	12.16	12.62	13.15
	0.16973	11.74	12.21	12.66	13.18
	0.19491	11.78	12.22	12.71	13.22
	0.23015	11.82	12.28	12.74	13.25
	0.27596	11.86	12.32	12.81	13.31
x_W = 0.200	0.02621	11.45	11.87	12.32	12.78
	0.04975	11.39	11.94	12.39	12.85
	0.07225	11.47	11.89	12.43	12.90
	0.12419	11.55	12.03	12.48	12.95
	0.16946	11.60	12.06	12.51	13.03
	0.19216	11.65	12.08	12.57	13.04
	0.23052	11.69	12.14	12.62	13.10
	0.27650	11.76	12.21	12.66	13.14
x_W = 0.100	0.02383	11.14	11.57	12.01	12.46
	0.05002	11.13	11.56	11.99	12.45
	0.07364	11.16	11.58	12.02	12.48
	0.12204	11.25	11.68	12.12	12.58
	0.17099	11.31	11.74	12.18	12.65
	0.19562	11.35	11.78	12.23	12.70

	0.23233	11.45	11.85	12.30	12.77
	0.27585	11.52	11.95	12.41	12.89
$x_W = 0.000$	0.02386	10.64	11.03	11.44	11.85
	0.05145	10.67	11.06	11.47	11.88
	0.07363	10.69	11.09	11.49	11.91
	0.12070	10.81	11.21	11.61	12.11
	0.17089	10.99	11.38	11.80	12.23
	0.19481	11.03	11.43	11.85	12.33
	0.23328	11.15	11.55	11.97	12.41
	0.27780	11.28	11.68	12.10	12.56

The uncertainty of the mole fraction x_W is equal to $\pm 1 \cdot 10^{-3}$. The uncertainty of molality is equal to $\pm 1.5 \cdot 10^{-5}$ mol·kg^{-1}.

Table S10. The apparent molar isentropic compression of hexaglyme in the DMF + W solvent.

		$K_{S,\Phi,m} \cdot 10^{14} / \left(m^3 \cdot Pa^{-1} \cdot mol^{-1}\right)$			
	m/(mol·kg^{-1})	293.15 K	298.15 K	303.15 K	308.15 K
$x_W = 1.000$	0.02654	–0.57	0.54	1.82	2.82
	0.04718	–0.64	0.58	1.76	2.74
	0.07348	–0.69	0.58	1.71	2.70
	0.12072	–0.72	0.52	1.62	2.66
	0.16577	–0.76	0.48	1.60	2.57
	0.19290	–0.77	0.44	1.55	2.54
	0.23377	–0.81	0.40	1.51	2.50
	0.26703	–0.84	0.38	1.47	2.47
$x_W = 0.980$	0.02630	0.76	1.73	2.88	3.59
	0.04688	0.77	1.73	2.77	3.67
	0.07142	0.81	1.72	2.74	3.60
	0.11931	0.73	1.70	2.72	3.58
	0.17341	0.69	1.65	2.66	3.51
	0.19399	0.67	1.63	2.63	3.51
	0.22823	0.63	1.60	2.61	3.47
	0.26482	0.61	1.57	2.58	3.42

x_W = 0.960	0.02806	1.23	2.26	3.11	3.93
	0.04993	1.30	2.34	3.07	3.88
	0.06868	1.32	2.33	3.11	3.88
	0.11870	1.41	2.43	3.14	3.97
	0.16749	1.52	2.51	3.23	4.00
	0.19471	1.57	2.54	3.25	4.03
	0.23961	1.66	2.62	3.32	4.08
	0.26936	1.70	2.66	3.35	4.11
x_W = 0.940	0.02764	2.40	3.03	3.84	4.66
	0.04917	2.42	3.21	3.92	4.73
	0.06764	2.54	3.22	3.98	4.82
	0.11687	2.79	3.47	4.17	4.95
	0.16487	3.03	3.69	4.37	5.14
	0.19164	3.16	3.82	4.47	5.25
	0.23578	3.41	4.03	4.66	5.41
	0.26501	3.56	4.17	4.78	5.51
x_W = 0.920	0.02492	3.43	4.28	4.96	5.65
	0.04866	3.56	4.24	4.93	5.64
	0.07143	3.59	4.28	4.99	5.71
	0.11787	3.68	4.38	5.05	5.74
	0.17385	3.80	4.47	5.13	5.80
	0.20086	3.85	4.52	5.17	5.85
	0.22911	3.91	4.57	5.23	5.88
	0.25937	3.96	4.63	5.28	5.92
x_W = 0.900	0.02702	4.36	4.97	5.61	6.09
	0.04911	4.36	5.02	5.62	6.22
	0.06985	4.45	5.08	5.67	6.28
	0.11608	4.58	5.24	5.81	6.39
	0.16915	4.73	5.39	5.96	6.52
	0.19264	4.79	5.46	6.03	6.56
	0.23327	4.93	5.58	6.14	6.69
	0.26404	5.02	5.67	6.23	6.75
x_W = 0.800	0.02631	7.82	8.34	8.74	9.16

	0.05015	7.85	8.31	8.80	9.24
	0.06965	7.92	8.37	8.79	9.25
	0.12002	7.99	8.42	8.87	9.32
	0.16883	8.04	8.49	8.93	9.39
	0.19503	8.07	8.51	8.95	9.41
	0.23372	8.13	8.58	9.01	9.48
	0.26945	8.17	8.62	9.06	9.53
x_W = 0.700	0.02721	10.21	10.70	11.23	11.60
	0.05019	10.30	10.81	11.26	11.64
	0.07042	10.32	10.78	11.27	11.71
	0.11807	10.43	10.89	11.33	11.75
	0.16552	10.49	10.95	11.40	11.84
	0.19639	10.52	10.98	11.45	11.88
	0.23262	10.58	11.05	11.50	11.95
	0.26930	10.64	11.11	11.57	12.00
x_W = 0.600	0.02582	11.72	12.10	12.50	12.94
	0.04807	11.67	12.15	12.56	13.00
	0.07046	11.73	12.18	12.58	13.10
	0.11933	11.81	12.23	12.69	13.13
	0.16988	11.88	12.29	12.73	13.21
	0.19224	11.92	12.33	12.77	13.25
	0.23002	11.96	12.39	12.86	13.31
	0.27268	12.04	12.47	12.91	13.38
x_W = 0.500	0.02620	12.33	12.77	13.22	13.71
	0.04908	12.36	12.80	13.37	13.86
	0.07135	12.39	12.91	13.37	13.86
	0.11924	12.47	12.96	13.43	13.92
	0.16660	12.54	13.01	13.52	14.01
	0.19558	12.57	13.07	13.57	14.07
	0.23001	12.62	13.12	13.62	14.12
	0.27069	12.69	13.18	13.68	14.18
x_W = 0.400	0.02533	12.69	13.14	13.60	14.10
	0.04807	12.67	13.12	13.57	14.07

	0.07303	12.70	13.15	13.69	14.20
	0.12068	12.77	13.27	13.74	14.24
	0.16770	12.86	13.31	13.78	14.29
	0.19931	12.90	13.35	13.82	14.33
	0.23383	12.96	13.39	13.87	14.38
	0.27474	13.02	13.46	13.94	14.45
$x_W = 0.300$	0.02667	12.72	13.19	13.68	14.18
	0.05097	12.66	13.13	13.62	14.12
	0.07071	12.72	13.18	13.67	14.18
	0.11946	12.74	13.21	13.70	14.20
	0.16816	12.84	13.31	13.80	14.31
	0.19414	12.88	13.32	13.81	14.32
	0.23318	12.93	13.38	13.87	14.39
	0.27451	12.99	13.47	13.94	14.45
$x_W = 0.200$	0.02406	12.35	12.79	13.26	13.77
	0.05074	12.33	12.90	13.38	13.88
	0.06912	12.39	12.83	13.40	13.91
	0.12024	12.49	12.94	13.41	13.98
	0.17291	12.58	13.03	13.51	14.06
	0.19690	12.64	13.09	13.57	14.08
	0.23388	12.70	13.16	13.63	14.15
	0.26538	12.77	13.20	13.68	14.19
$x_W = 0.100$	0.02556	11.98	12.40	12.87	13.35
	0.04951	12.14	12.56	13.04	13.52
	0.07371	12.11	12.54	13.01	13.49
	0.12028	12.22	12.65	13.13	13.61
	0.16628	12.37	12.77	13.25	13.74
	0.19362	12.42	12.87	13.31	13.80
	0.23091	12.52	12.95	13.43	13.92
	0.28006	12.64	13.08	13.54	14.04
$x_W = 0.000$	0.02672	11.48	11.90	12.31	12.78
	0.04932	11.61	12.03	12.45	12.92
	0.07293	11.68	12.11	12.53	13.00

0.12086	11.79	12.22	12.71	13.18
0.17102	11.97	12.45	12.88	13.36
0.19284	12.07	12.51	12.95	13.42
0.23187	12.23	12.67	13.12	13.60
0.28013	12.41	12.85	13.31	13.79

The uncertainty of the mole fraction x_W is equal to $\pm 1 \cdot 10^{-3}$. The uncertainty of molality is equal to $\pm 1.5 \cdot 10^{-5}$ mol·kg^{-1}.

NMR Spectroscopy.

NMR spectra were recorded using a Bruker AVANCE III spectrometer equipped with a 5 mm TBI probe at 600.13 MHz. The ^{1}H chemical shifts (δ in parts per million) were specified with respect to the $CDCl_3$ (7.26 ppm) signal as a reference. The 1D ^{1}H NMR spectra were measured at 302 K by means of the 90° single-pulse experiment (Bruker pulprog: zgig) with ^{13}C ‘garp’ decupling mode. The 90° pulse width for each sample was determined by prorating the measured 360° pulse width (p_{90} = ¼ x p_{360}). The gain of the receiver was set to 45 for all ^{1}H NMR measurements. A total number of 16 transients recorded at an acquisition time of 3.0 s and a relaxation delay of 24 s, (5 times the longest T_1 observed) was obtained The other parameters were: spectral width 12500.00 Hz, FID resolution 0.17 Hz. Frequency was tuned and impedance matched before each measurement run. The 1D NMR data analysis was performed using TopSpin software (v.2.1 p4 , Bruker BioSpin). For ^{1}H NMR spectra analysis, standard Furier transform (128 k digital data point). without line broadening and Gaussian factor (line broadening = 0 Hz, Gaussian factor = 0) was applied, The digital resolution was 0.095 Hz/pt (0.22 ppb/pt at 600.13 MHz). A fifth-order polynomial baseline correction was applied, after manual phasing. Dimethyl terephthalate (Sigma-Aldrich, TraceCert®) was used as internal standard.

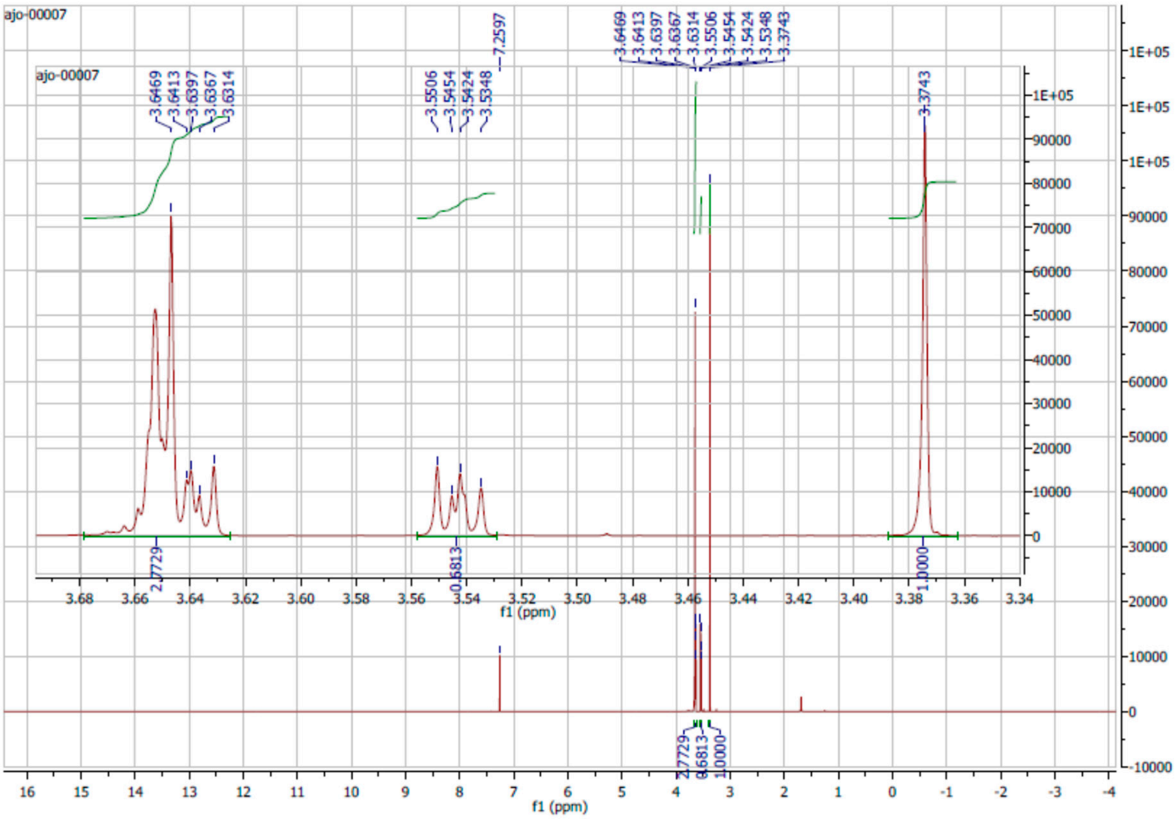

pentaglyme

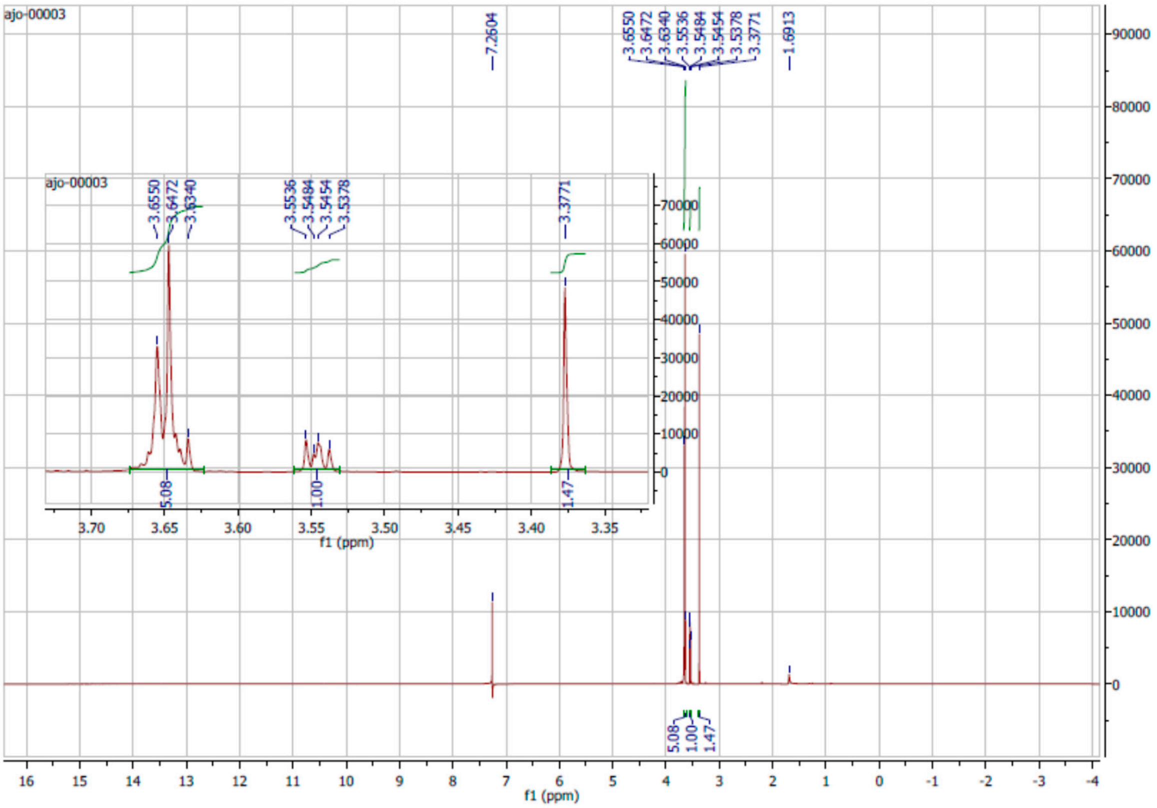

heksaglyme
